# Genetic Divergence of H1N1pdm09 in Saudi Arabia: Unveiling a Novel N-Glycosylation Site and Its Role in Vaccine Mismatch

**DOI:** 10.3390/vaccines13111111

**Published:** 2025-10-30

**Authors:** Shatha Ata Abdulgader, Abdulhadi M. Abdulwahed, Abdulaziz M. Almuqrin, Ibrahim M. Aziz, Noorah A. Alkubaisi, Reem M. Aljowaie, Mohamed A. Farrag, Abdulkarim F. Alhetheel, Adel A. Abdulmanea, Fatimah N. Alanazi, Asma N. Alsaleh, Fahad N. Almajhdi

**Affiliations:** 1 Department of Clinical Laboratory Sciences, College of Applied Medical Sciences, King Saud University, Riyadh 12372, Saudi Arabia; 444204318@student.ksu.edu.sa (S.A.A.); aabdwahed@ksu.edu.sa (A.M.A.); aalmuqrin@ksu.edu.sa (A.M.A.); 2 Department of Botany and Microbiology, College of Science, King Saud University, P.O. Box 2455, Riyadh 11451, Saudi Arabia; iaziz@ksu.edu.sa (I.M.A.); raljowaie@ksu.edu.sa (R.M.A.); mfarrag@ksu.edu.sa (M.A.F.); aabdulmanea@ksu.edu.sa (A.A.A.); 445205516@student.ksu.edu.sa (F.N.A.); asmalsaleh@ksu.edu.sa (A.N.A.); 3 Department of Pathology and Laboratory Medicine, College of Medicine, King Saud University, Riyadh 11451, Saudi Arabia; aalhetheel@ksu.edu.sa

**Keywords:** influenza A virus, A/H1N1pdm09, vaccine effectiveness, phylogenetic analysis, glycosylation, Saudi Arabia, molecular epidemiology

## Abstract

**Background/Objectives:** Influenza A virus undergoes continuous antigenic drift, necessitating annual vaccine reformulation. Saudi Arabia faces unique epidemiological challenges owing to mass gatherings during religious pilgrimages and the dynamic movement of foreign workers. This study aimed to characterize the genetic diversity of hemagglutinin (*HA*) and neuraminidase (*NA*) genes of influenza A viruses circulating in Riyadh and to assess their match with vaccine strains during the 2024–2025 period. **Methods**: Nasopharyngeal samples (n = 363) were collected from patients presenting with influenza-like illness. RT-PCR was used for detection and subtyping. Sequence and phylogenetic analysis of the complete *HA* and *NA* gene sequences from A/H1N1pdm09 strains (n = 7) were then performed. **Results**: Of the 363 samples, 110 (30.3%) were positive for influenza A; among these, 68 (61.8%) were A/H1N1pdm09, and 42 (38.2%) were H3N2. Phylogenetic analysis revealed that all A/H1N1pdm09 strains belonged to clade 5a.1, distinct from vaccine strains. In comparison with the vaccine strain A/Wisconsin/67/2022, seven amino acid substitutions in the *HA* gene and eight in the *NA* gene were recorded in Saudi circulating strains. The significant genetic divergence between circulating A/H1N1pdm09 strains and current vaccine strains indicates potential vaccine mismatch. **Conclusions**: The significant genetic divergence between circulating A/H1N1pdm09 strains and current vaccine strains suggests potential vaccine mismatch. Continuous surveillance programs along with vaccination plans are necessary to tackle the changing influenza A virus strains in the special epidemiological context of Saudi Arabia.

## 1. Introduction

Influenza A virus remains a major global health threat. The catastrophic 1918 pandemic caused an estimated 20 to 40 million deaths [1]. The World Health Organization (WHO) estimates that around 1 billion instances of human influenza occur, with 3–5 million people suffering from severe infections, particularly children, the elderly, and immunocompromised individuals. The virus causes significant morbidities and mortalities, recording 290,000 to 650,000 deaths annually [2,3]. According to the International Committee on Taxonomy of Viruses (ICTV), influenza viruses are members of the *Orthomyxoviridae* family, which are characterized by having a negative-sense, segmented RNA genome. The family comprises nine genera, with influenza viruses falling into four genera, *Alpha*, *Beta*, *Gamma*, and *Deltainfluenzavirus*. The first two genera are clinically significant for humans [4]. Viruses of the genus *Alphainfluenzavirus* are classified into subtypes based on the antigenic variations present in their hemagglutinin (*HA*) and neuraminidase (*NA*) antigens [5]. Currently circulating subtypes in humans are A/H1N1 and A/H3N2 influenza viruses [6].

During the 20th century, these subtypes caused four pandemics. Apart from the Spanish flu mentioned previously, significant outbreaks include the Hong Kong flu (A/H3N2) in 1968, and the Asian flu (A/H2N2), which both resulted in high mortality rates. The A/H1N1 flu pandemic of 2009, which originated from pigs and was first identified in Mexico, impacted more than 60.8 million individuals globally [7]. In Saudi Arabia, by December 2009, there were 15,850 confirmed laboratory cases and 124 reported deaths of the disease [8].

*HA* and *NA* are principal glycoproteins of influenza viruses and are responsible for binding to cell receptors and release from host cells, respectively. *HA* is a complex molecule that is formed of three monomers synthesized as inactive precursor protein HA0, which is cleaved into HA1 and HA2 subunits by cellular proteases and is a prerequisite for viral infectivity in humans. HA1 is the receptor-binding domain that attaches to sialic acid moieties on host cells, while HA2 is membrane-anchored and is responsible for fusion to the host cell membrane [9]. Changes in the amino acid residues of HA1 can cause alteration of the influenza virus binding preference. They may confer resistance to the strain-specific antibodies that target the globular head of the *HA* protein [10]. Also, it may cause a mismatch between the current vaccine and the circulating strain of influenza. Consequently, annual surveillance of the influenza virus is crucial for gathering vital information necessary for the reformulation of yearly influenza vaccines and detecting any possible epidemic and pandemic [11]. In Saudi Arabia, the desert climate is considered one of the primary factors influencing the transmission of influenza. Studies on influenza from Saudi Arabia can inform public authorities and clinical practitioners about the molecular epidemiology of the influenza virus. However, several investigations have documented influenza A (H1N1) virus circulation in different regions of Saudi Arabia. For example, cases were reported in a tertiary care hospital in the Khamis Mushyt region between July 2009 and June 2010, where clinical characteristics of hospitalized patients were described [12]. Utilizing RT-PCR, Uthman et al. detected A/H1N1pdm09 infection in only 10 out of 100 clinical samples [13]. More recently, during 2020–2022, 21 out of 200 clinical samples (10.5%) from hospitalized children in Riyadh were positive for influenza A virus, with 71.4% of isolates identified as A/H1N1pdm09 [14]. In one study conducted in King Fahad Medical City in Riyadh from 2014 to 2015, 149 samples of hospitalized patients with flu-like symptoms were included. The study reported 80 positive samples of A/H1N1, and phylogenetic analysis showed similar topologies and co-circulation of clade 6 B. Also, mutations at key antigen sites, such as S101N and S179N (at antigenic site-Sa) and I233T (at antigenic site-Sb) in the head domain, suggested antigenic drift and the emergence of variant viruses in the study [15]. A similar finding was reported in a previous study [16].

Despite studies conducted in Riyadh on the influenza virus, few have investigated the epidemiology and genetic variation in the influenza virus during the winter season 2024–2025. Therefore, this study aimed to identify the epidemiology, genetic changes, and evolutionary kinetics of influenza A virus, especially type A/H1N1, circulating in Riyadh, Saudi Arabia. We hypothesized that circulating strains would show significant divergence from vaccine strains due to ongoing antigenic drift.

## 2. Materials and Methods

### 2.1. Ethics Statement

This study was performed at the Virology Laboratory, Botany and Microbiology Department, College of Science, King Saud University. The study was conducted in accordance with the Declaration of Helsinki and approved by the Research Ethics Committee at King Saud University in Riyadh, Saudi Arabia (Institutional Review Board No. 22/0957/IRB and Ref. No. 22/0957/IRB, approved 27 November 2023 and 18 May 2025, respectively). Samples positive for influenza A virus identified during the study were managed in strict adherence to ethical guidelines, ensuring patient confidentiality and appropriate medical follow-up as per King Saud University’s protocols.

### 2.2. Patient Selection and Clinical Samples

The samples were collected according to the protocol of Influenza Surveillance in Saudi Arabia, which stated that any patient presenting with fever, cough, sore throat, runny nose, muscle or body aches, headache, and fatigue was considered to be typical influenza symptoms [17]. A total of 363 samples were gathered from hospital wards and emergency rooms at King Khalid University Hospital. The samples involved throat swabs, nasopharyngeal aspirates (NPAs), and sputum, which were collected during the epidemic year of 2024–2025. Upon collection, the samples were combined with 2 mL of virus minimum essential medium (MEM) transport medium (Gibco, Invitrogen, Grand Island, NY, USA). They were then transported under refrigerated conditions to the Virology Research Laboratory at King Saud University’s College of Science, where they were stored at −80 °C until further analysis.

### 2.3. Identification, Typing, and Sequencing of Influenza a Virus

#### 2.3.1. Identification and Typing

Extraction of viral RNA from clinical samples was carried out according to the manufacturer’s instructions of QIAamp Viral RNA Extraction Kit (Qiagen, Hilden, Germany). By utilizing the universal primer One-Step Ahead RT-PCR Kit with Taq High Fidelity DNA Polymerase (Qiagen, Hilden, Germany, cat. no. 220213), the detection of influenza B virus was achieved. The GeneAmp 9700 thermal cycler was used to perform the reaction under the following cycling conditions. Reverse transcription was conducted at 50 °C for 30 min. This was followed by an initial denaturation phase at 95 °C for 15 min. The cycling program included 35 cycles comprising denaturation at 4 °C for 30 s, annealing at 52 °C for 30 s, and extension at 72 °C for 2 min. A final extension step was performed at 72 °C for 10 min. By using agarose gel stained with 1% ethidium-bromide, the PCR products were visualized under UV light and compared to 100 bp Plus DNA ladder (Qiagen, Hilden, Germany).

#### 2.3.2. Sequencing and Editing of A/H1N1 pdm09 Strains

The antigenic glycoprotein genes of A/H1N1 pdm09, including the *HA* gene and the *NA* gene, were both amplified using the same amplification kit and two sets of overlapping primers. The product was then imported into a GeneAmp 9700 thermal cycler to complete the amplification process and achieve a complete sequence of *HA* and *NA*. Details regarding the detection, typing, and sequences of the primers employed in the PCR are provided in Table 1.

The resulting amplified *HA* (1701 bases) and *NA* (1410 bases) gene fragments were sequenced in both directions at Macrogen Inc. (Seoul, Republic of Korea). Raw sequence data were edited using the BioEdit program, version 7.0 (Ibis Biosciences, Carlsbad, CA, USA), and assembled by the Edit sequence tool of the MegAlign program, Lasergene software, version 3.18 (DNAStar, Madison, WI, USA). Only 7 sequences were selected for sequence and phylogenetic analysis based on their sequence heterogeneity. The 7 samples out of 68 positive A/H1N1pdm09 strains that were analyzed only represent a fraction of the total positive samples. We do, however, emphasize that these 7 strains were the ones that successfully underwent amplification and sequencing of both *HA* and *NA* genes; this process is frequently the bottleneck in molecular surveillance studies, especially when dealing with clinical samples that might have different viral loads and RNA integrity. The final sequences in this study have been deposited in GenBank with accession numbers PV652976 to PV652982 for the *HA* gene and PV653584 to PV653590 for the *NA* gene.

### 2.4. Sequence and Phylogenetic Analysis

The sequences of *HA* and *NA* genes were aligned using the Clustal W method with a total of 83 international circulating strains of A/H1N1pdm09, which were retrieved from the GISAID database and the Gene Bank website. Alignments were performed against the prototypic A/H1N1pdm09 reference A/California/7/2009 (GenBank: NC_026433 for HA; NC_026434 for NA) to highlight evolutionary divergence from the pandemic origin. For divergence analysis, mutation site identification, and the prediction of amino acid changes, the EditSeq and MegAlign programs, along with Lasergene software version 3.18 (DNAStar Inc., Madison, WI, USA), were employed. The phylogenetic analysis incorporated sequences from reference strains of established clades and vaccine strains suggested by the WHO, which were retrieved from the GenBank and GISAID databases (see Table S1). The assessment of heterogeneity in N-glycosylation (Asn-X-Ser/Thr, with X representing any amino acid except proline) and O-glycosylation sites (Ser or Thr) within the HA and NA proteins of A/H1N1 strains was conducted using NetNGlyc 1.0 [18] and NetOGlyc 3.1 [19]. The phylogenetic analysis was executed using the neighbor-joining method in MEGA 11 (v.11, Pennsylvania State University, University Park, PA, USA), with 1000 bootstrap replications performed. Bootstrap values exceeding 60% were marked on the principal tree branches for clarity.

### 2.5. Statistical Analysis

Statistical analysis was performed using IBM SPSS Statistics v26.0 (IBM Corp., Armonk, NY, USA). Categorical variables were evaluated via Fisher’s exact test, with post hoc *Z*-tests adjusted by Bonferroni correction. A significance level of *p* < 0.05 was considered indicative of statistical significance.

## 3. Results

### 3.1. Identifying and Typing of the Influenza a Virus

Based on the year of collection, samples were divided into two subgroups: one for 2024 and the other for 2025. A/H1N1pdm09was the most prevalent virus in 2024 (80.8%), although A/H3N2 increased gradually in 2025 (52.3%). Of the total 363 clinical samples included in the study during the epidemic season of 2024 and 2025, 176 were males and 187 were females. Only 110 (30.3%) samples were positive for influenza A virus. Among these positive samples, the A/H1N1pdm09strain was predominant compared to A/H3N2 strains, with numbers reaching 68 (61.8%) and 42 (38.1%), respectively. Age groups were divided based on individuals who are at risk for developing complications of influenza [20]. Four consolidated groups were established: 0–4 years, 5–14 years, 15–64 years, and 65 years and older. Notably, people aged 15–64 years represented the highest impact, accounting for 32.6% of the overall sample size. Regarding the results depending on gender, females were more affected (35.2%) compared to males (25.5%), as shown in Table 2.

### 3.2. Analysis of Nucleotide and Amino Acid Sequences of the HA and NA Genes in A/H1N1pdm09 Subtype Study Strains

**Figure 1:**
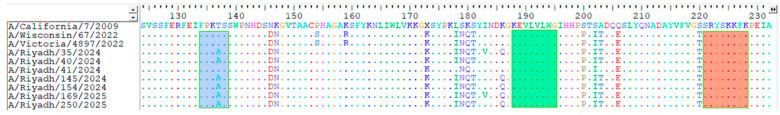
Alignment of the deduced amino acid sequences of the *HA* gene. The alignment was performed using the Bioedit program, version 7.2. Dots indicate identical residues and different residues are shown in a single-letter code. The 130-loop (residues 134–138) is represented by the enclosed blue rectangle; the 190-helix (residues 188–195) is represented by the enclosed green rectangle; the 220-loop (residues 221–228) is represented by the enclosed red rectangle.

The sequence analysis revealed that nucleotide identity ranged from 97.5 to 98.2% (HA) and 96.8–97.9% (NA). Mutation analysis showed the presence of 67 mutation sites in the *HA* gene of A/H1N1 strains. 27 of these mutations might lead to amino acid changes, and only two mutations were identified as unique (K186Q and Q406L) compared to other international strains (Figure S1A–D). In comparison with the reference strain A/California/7/2009 and the vaccine strains (A/Wisconsin/67/2022 and A/Victoria/4897/2022), we identified several antigenicity-related homologous sequences in a receptor-binding domain (RBD) in the *HA* gene of Riyadh strains, specifically the 130-loop (residues 134–138), the 190-helix (residues 188–195), and the 220-loop (residues 221–228) (Table 3) and (Figure 1). For the *NA* gene, a total of 62–70 nucleotide mutation sites were detected, 27 were reported to cause amino acid changes. Of these, only two unique substitutions (S12F and S52N) were reported in our strains but not found in the international strains (Figure S2A–D).

**Figure 2:**
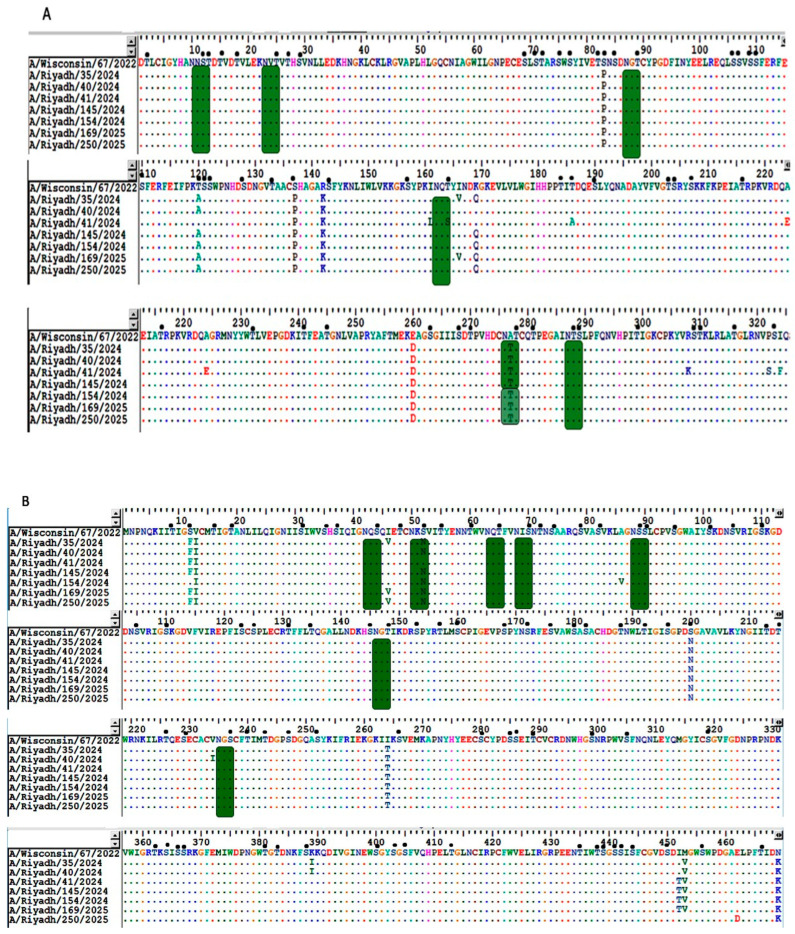
Alignment and comparison between A/Riyadh A/H1N1pdm09 strains and vaccine strain (A/Wisconsin/67/2022), (**A**) *HA* gene, and (**B**) *NA* gene. Similar amino acids are represented by colored dots, while the amino acid changes occurred and are shown in capitalized alphabetical order. Location of N-glycosylation site indicated in green rectangles and possible O-glycosylation site marked by black dots.

For the aim of this study, a comparison was performed between the HA1 domain of the current vaccine strain of A/H1N1 (A/Wisconsin/67/2022) and study strains of A/Riyadh. The analysis revealed that the HA1 domain of our A/H1N1pdm09strains had seven amino acid substitutions: S38P, T120A, S137P, R142K, K169Q, I260V, K142D, and A277T. These mutations were unique to our strains as compared to the vaccine strain, as depicted in Figure 2A. The identical analysis was carried out on the *NA* gene of our A/H1N1pdm09 strains. A total of 8 amino acid substitutions were identified, and the majority of these mutations were not reported in the current vaccine strain of A/H1N1pdm09. A description of these *NA* gene mutations is shown in Figure 2B.

### 3.3. Analysis of N and O Glycosylation Sites

In comparison with the prototype A/H1N1pdm09 (reference A/California/7/2009), HA protein of Riyadh A/H1N1 pdm09 showed six N-glycosylation sites (27 NNS, 40 NVT, 87 NGT, 293 NTT, 304 NTS, and 499 NGT). In contrast, O-glycosylation sites ranged from 64 to 68 sites, as observed in (Figure S1A–D).

The examined open reading frame of the HA1 protein from A/H1N1 pdm09 sequences contains 6 N-glycosylation sites (10 NNS, 23 NVT, 87 NGT, 162 NQT, 276 NAT, and 287 NTS). All identified potential N-glycosylation sites were documented in vaccine strains and were also observed in the strains analyzed in this research. In contrast, the HA1 domain exhibits significant glycosylation with O-linked carbohydrates attached to serine and threonine residues. The potential O-linked glycosylation sites found in the vaccine strain A/Wisconsin/67/2022 ranged from 48 to 55. Compatible O-glycosylation sites were also detected in all strains studied, as illustrated in Figure 2A.

The prototype H1N1pdm09 reference A/California/7/2009 and the NA protein of Riyadh A/H1N1 pdm09 showed seven N-glycosylation sites (58 NNT, 63 NQT, 86 NIS, 88 NSS, 148 NGT, and 235 NGS). Additionally, one strain included in this investigation has been shown to have an additional potential N-glycosylation site that was shown in the prototype A/H1N1pdm09 reference A/California/7/2009. This site is located at position 70, where the amino acid (S) was substituted with (N) (N50N), resulting in the absence of the N-glycosylation site (50 NQS). Conversely, as shown in (Figure S2A–D), O-glycosylation sites varied from 85 to 90 sites.

In the *NA* gene of the study strain A/H1N1pdm09 and vaccine strain (A/Wisconsin/67/2022) sequences, the N-glycosylation sites are predicted to contain 7 sites (42 NQS, 50 NKS, 63 NQT, 68 NIS, 90 NSS, 146 NGT, and 235 NGS). All of the 7 N-glycosylation sites found in the study strains were also identified in the vaccine strain. In contrast, O-glycosylation sites ranged between 76 and 81 sites, as observed in Figure 2B.

### 3.4. Phylogenetic Analysis

**Figure 3:**
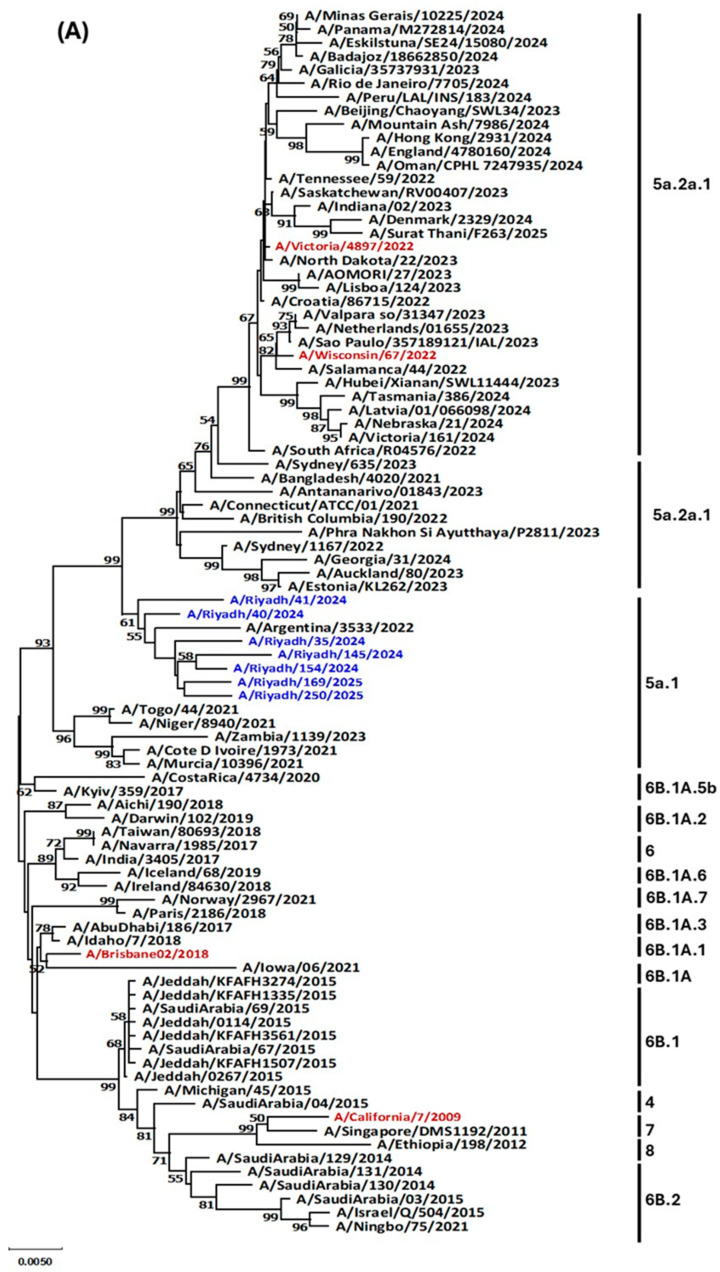

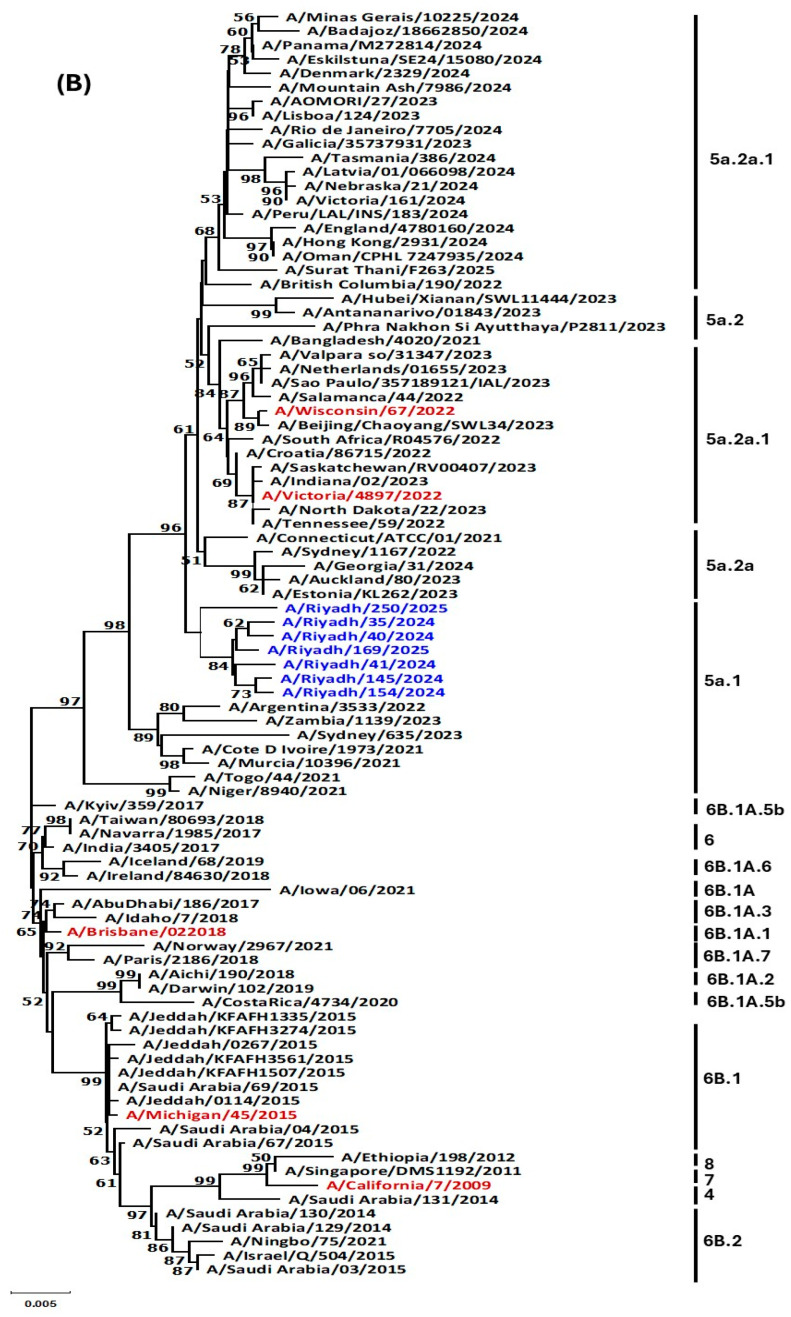
A/H1N1pdm09phylogenetic tree based on nucleotide sequences according to the (**A**) *HA* gene and (**B**) *NA* gene. A/H1N1pdm09study strains are colored in blue, vaccine strains are denoted in red.

To analyze genetic variation between different strains, phylogenetic analysis for both *HA* and *NA* genes was conducted. This analysis included A/H1N1pdm09 study strains, the vaccine strain recommended by WHO for 2024–2025, and reference, local, and global strains retrieved from GenBank and GISAID. As shown in Figure 3A,B, all A/H1N1pdm09 study strains (A/Riyadh/35/2024, A/Riyadh/40/2024, A/Riyadh/41/2024, A/Riyadh/145/2024, A/Riyadh/154/2024, A/Riyadh/169/2025, A/Riyadh/250/2025) were clustered in group 5a.1. Furthermore, all strains showed sequence homology with A/Argentina/3533/2022 strain with varying percentages ranging from 47 for (A/Riyadh/35/2024, A/Riyadh/145/2024, A/Riyadh/154/2024, A/Riyadh/169/2025, A/Riyadh/250/2025) and 55% for (A/Riyadh/40/2024, A/Riyadh/41/2024). Interestingly, the results demonstrated that A/A/H1N1 isolates were not closely related to any influenza vaccine strains listed in phylogenetic analysis.

## 4. Discussion

The seasonal incidence of influenza viruses requires ongoing surveillance to identify new variants that may lead to human pandemics. This monitoring also assists health authorities in determining which viral strains should be incorporated into seasonal vaccines [21]. In Saudi Arabia, the number of such reports concerning the epidemiology of the influenza virus has increased since 2009; however, little is known about the circulating strain of influenza in Riyadh during the year 2024–2025. As such, the present research sought to determine the subtypes of influenza A virus and evaluate their epidemiological traits, genetic variation, and evolutionary dynamics in Riyadh over one year (2024–2025), to guide policy decisions regarding influenza vaccination.

In the current study, of the 363 samples collected, 110 were positive for influenza A virus, with type A/H1N1 pdm09 having a distinct predominance of 38 (80.0%) compared to 9 (19.1%) H3N2 samples during the year 2024 in Riyadh. These findings align with an our an earlier investigation which reported a high number of circulating H1N1 strains compared to H3N2 during five epidemiological seasons (2016–2020) [17]. Similarly, a prior investigation conducted across 15 nations in the Eastern Mediterranean Region identified confirmed influenza cases in patients exhibiting signs of severe acute respiratory infection. The study found that the A/H1N1pdm09subtype was the most commonly circulating variant, accounting for 41.7% of cases, while the A/H3N2 subtype followed with 671 instances [22]. In contrast, the circulation of H3N2 steadily rose until it overcame the number of instances for A/H1N1 by 52.8%. These results are similar to those found in the research developed by Al-Dorzi et al. (2024) [23]. The number of circulating strains A/H3N2 increased during the epidemic year of 2021–2022, accounting for 244 cases compared to 151 of the A/H1N1 strain [23]. Also, Al-Baadani et al. (2019) [24] reported that among 448 collected samples, 216 were positive for A/H3N2 and 150 were A/H1N1. These inconsistencies in the circulation patterns might be due to the high mutation rate of the A/H3N2 strain and its relation with the occurrence of local epidemics [25].

Influenza virus complications are associated with the age of infected individuals. Indeed, infants under the age of 5 and people aged more than 65 are at elevated risk for influenza complications [26]. A study reported that during the pandemic, the A/H1N1pdm09 virus predominantly impacted children and adolescents more than older adults. This is demonstrated by the significant disparity in seroprevalences before and after the pandemic, along with a higher incidence rate of microbiologically confirmed infections specific to age, and an elevated positivity rate in respiratory tract samples compared to other respiratory viruses. Notably, individuals aged 51 to 60 represented the largest cohort of severe cases, as this group also exhibited the highest rate of severe occurrences. Furthermore, the analysis of positivity rates in respiratory tract specimens indicates that the A/H1N1pdm09 virus was the leading cause of respiratory infection among individuals aged 50 and younger [27]. Herein, we report that people aged 15–64 had the highest impact, accounting for 32.6% of the overall sample size. Also, we found that females were more affected (35.2%) than males (25.5%). Our findings are in parallel with a previous study conducted at Riyadh in King-Fahad Medical City, which reported that the median age of influenza-infected was 31. The majority of them were female (66%) [28]. Also, another study in western Saudi Arabia found that the most infected age group was (19–60), and females comprised half of the patients [29].

In earlier studies, the globular head region of the HA protein in A/H1N1 viruses was shown to contain five antigenic sites: Ca1, Ca2, Cb, Sa, and Sb. A single amino acid change can significantly affect the virus’s ability to provoke an immune response and the effectiveness of vaccines in safeguarding these essential antigenic sites [30].

In our study, influenza A/H1N1pdm09-specific primer set H1F1 (5′-AGCAAAAGCAGGGGAAAATAAAAGC-3′) and H1F2 5′-GGGAGAATGAACTATTACTGG was used to amplify the full length *HA* gene of A/H1N1 pdm09 [2]. The findings of this study align with those of an earlier studies which used the same primers for influenza A/H1N1 pdm09 with the prototypic A/H1N1pdm09 reference A/California/7/2009 [14,31,32]. The positive reaction of our samples with this primer set confirms that our strains originated from the pandemic strain. In addition, sequence alignments revealed that nucleotide identity ranged from 97.5 to 98.2% (*HA* gene) and 96.8–97.9% (*NA* gene), which confirm the pandemic origin of our strains.

The phylogenetic analysis showed that all study strains are circulating in the same clade as A/Argentina/3533/2022, A/Togo/44/2021, and A/Niger/8940/2021 5a.1. This clade was the same reported among circulating strains in Riyadh during the epidemic years of 2020–2021 and 2021–2022 [14]. In contrast, another study found different results for the clade of the circulating A/H1N1 in Riyadh during the epidemic year of 2021–2022, as the reported clade was 5a.2a [21]. Reasons for these differences might include the fact that multiple introductions of viruses significantly influence evolution within local epidemics, facilitating the simultaneous circulation of various clades belonging to the same subtype [33,34]. On a global level, the movement of viruses from areas with more sustained influenza transmission, particularly in East and South-East Asia, plays a crucial role in shaping extensive epidemiological trends [35,36,37]. Furthermore, reassortment events among viruses of the same subtype occur regularly and are occasionally linked to significant antigenic shifts in both the A/H3N2 [38], and A/H1N1 subtypes. Phylogenetic analysis also revealed that neither the vaccine strain nor the regional strains of A/H1N1 showed any genetic relatedness to the study A/H1N1pdm09 strains, which indicates that travel could have played a role in the dissemination of the virus [17]. In addition, it could also represent the vaccine’s incapability to provide optimal immunity, despite the WHO’s continuous efforts throughout the year to provide guidance on which vaccine strain should be included in both the Northern and Southern Hemispheres [39].

In this study, identifying the relevant reference strains was necessary to contextualize the evolutionary history of the present-day A/H1N1pdm09 viruses. For our phylogenetic and mutational assessments, we selected A/California/7/2009 as the baseline reference. A/California/7/2009 is the progenitor of the A/H1N1pdm09 and the ancestor of all A/H1N1pdm09 lineages, including the clade 5a.1 virus examined in this study. This is corroborated by decades of global sequencing data available through GISAID and GenBank [33]. In pandemic flu research, using this ancestor strain as an international reference is standard and helps in the tracking of mutations over time, offering comparisons to clades across different psoriasis [14,17]. This method helps to reconstruct the axis of the mutant virus’s evolution over the past decade.

A foundational reference is important to see how our isolates stack up against recent antigenically relevant strains. The concern with only aligning our sequences with hyper-homologous recent strains, like A/Argentina/3533/2022, is that it might mask genetic distances from the larger evolutionary perspective. However, it is useful to frame the recent microevolutionary perspectives. This is why we provided the A/Argentina/3533/2022 and other recent strains comparative analysis in Figure S1 as an additional perspective. For our evaluation of vaccine mismatch, we used A/Wisconsin/67/2022 (clade 5a.2a.1) as the comparator due to its direct public health relevance because it is the WHO-recommended vaccine component for the 2023–2025 Northern Hemisphere influenza season.

One effective method to prevent the spread of influenza is vaccination. Current seasonal influenza virus vaccines are designed to provoke neutralizing antibody responses specific to the *HA* head. However, the *HA* head domain exhibits considerable flexibility, making it susceptible to mutations that facilitate antigenic drift as mentioned before. Potential mitigation strategies for influenza A virus include developing next-generation vaccines (mRNA, viral vector, universal) that target conserved viral proteins, implementing a One Health plan with improved biosecurity and surveillance, and employing antiviral drugs. Other strategies include bolstering existing immunization platforms, personal protective equipment, and public health efforts. Adjuvanted vaccines, which contain ingredients like MF59, AS03, or alum, are a crucial tactic to increase the effectiveness of influenza A virus vaccines by strengthening the immune system, especially in susceptible populations like the elderly [40]. Since the production of vaccines must commence several months before the influenza season to ensure an adequate supply, the circulating viruses may differ from the vaccine strains. Additionally, the manufacturing process for seasonal influenza vaccines predominantly relies on eggs, which can lead to adaptive mutations in the virus, thereby reducing vaccine efficacy [41]. For influenza A virus and influenza B virus vaccines, the WHO organizes and hosts vaccine strain consultation meetings twice each year to select human vaccine strains for the Northern or Southern Hemispheres [42]. For this reason, the WHO provides more than 28 strains to ensure a high protection against the virus [17]. In Saudi Arabia, several strains have been provided by the Saudi Ministry of Health, including A/Victoria/4897/2022 (H1N1) pdm09-like virus, A/Wisconsin/67/2022 A/(H1N1) pdm09-like virus, and an A/Sydney/5/2021 (H1N1) pdm09-like virus. In this study, only four A/H1N1 vaccine strains were used as reference. To ensure the efficacy of the current vaccine against the circulating A/H1N1 pdm09 strains in Riyadh, the seven study strains were compared to the vaccine strain A/Wisconsin/67/2022. Seven and eight amino acid site substitutions were identified in the *HA* and *NA* genes of our A/H1N1pdm09 strains, respectively, that were not found in the current vaccine strain of A/H1N1 A/Wisconsin/67/2022. For the HA protein, our analysis revealed that the 7 amino acid changes are located within essential antigenic sites, specifically within the HA1 subunit, which is responsible for receptor binding and is a primary target for neutralizing antibodies. Modifications in these areas can significantly alter the antigenicity of the virus and thus help the virus to escape the immune system. The antigenic divergence we observed is also underscored by the 97.1–97.4% HA protein identity between our study’s strains and this vaccine virus, which, with the rapidly evolving nature of the virus, reflects the challenge of vaccine mismatch.

Vaccination minimizes the risk of flu illness when most circulating flu viruses are appropriately matched to the vaccine. However, the phenomenon of antigenic drift, which consistently takes place over time as the virus replicates, could reduce the effectiveness of the vaccine. The majority of influenza vaccinations are formulated to focus on the HA surface proteins or antigens of the influenza virus. Similarly to other subtypes, the pdm09 HA is crucial for attaching to host cell receptors and facilitating the release of viral RNA into the cell [43]. The structure includes the RBD within a globular head domain, which consists of three distinct domains: the 130-loop (residues 134–138), the 190-helix (residues 188–195), and the 220-loop (residues 221–228). This structure specifically targets sialic acid residues present on host cells [44,45,46]. Four key canonical epitopes, Ca (which includes Ca1 and Ca2), Cb, Sa, and Sb, have been recognized by the host immune system on the globular HA head. Changes in HA protein associated with antigenic drift occur continuously over time as the virus replicates to prevent them from being inhibited by antibodies generated by vaccination and natural infection [30]. Influenza viruses develop escape mutants that show variations in the structure and biological functions of HA, particularly in relation to the RBD. These mutants can cause reinfection in humans [43,47].

In the current study, N- and O-glycosylation sites identified for both gene *HA* and *NA* were also observed in the vaccine strain. Variations in the N- and O-linked glycosylation of the HA and NA proteins can influence the host specificity, pathogenicity, and infectivity of an influenza strain. This occurs by modifying the biological properties of HA and NA or by diminishing receptor binding and obstructing antigenic regions of the protein. Previous research has shown that seasonal viruses possess a greater number of glycosylation sites on the head of the HA and NA compared to A/H1N1 pdm09. Additionally, it was found that two glycosylation sites (50 and 68) on the stalk of the NA in A/H1N1 pdm09 may have been replaced by two other glycosylation sites (44 and 70) in seasonal strains. Recent studies further suggest that the glycosylation of the HA globular head domain acts to shield the antigenic sites, thereby hindering antibody recognition and facilitating the virus’s escape from antibody-mediated neutralization [14].

The primary limitation of this study is its small cross-sectional sample size. Furthermore, it does not establish a cause for the recurrence of infections. To gain a more comprehensive insight into the circulation patterns of A/H1N1pdm09, further rigorous research with larger sample sizes distributed across various regions of Saudi Arabia, including the big cities, during successive epidemic seasons, is necessary. In addition, hemagglutination inhibition (HI) assays should be performed to better understand the influence of genetic alterations on the vaccines’ efficacy.

## 5. Conclusions

In summary, this study reported the prevalence, genetic variation, and vaccine strain mismatch in Riyadh. The prevalence of influenza A reported in this study was 68% and 42% for A/H1N1pdm09 and A/H3N2, respectively. The A/H1N1pdm09 strains were predominant during 2024, while A/H3N2 rose steadily during 2025. People aged 15–64 and older were the most affected group (32.6%), with the highest number of positive samples, and the majority were females. Seven mutation sites have been detected in the HA domain of A/H1N1pdm09 strains, and eight amino acid changes occurred in the *NA* gene that were not reported in the current vaccine strain of A/H1N1pdm09. To confirm the importance of these substitutions and assess the vaccine’s efficacy on an annual basis, further local and regional studies will be crucial.

## Figures and Tables

**Table 1:** The primers list of A/H1N1 used in this study.

	Type/Subtype	Gene	Primer Name	Sequence 5′-3′	Product Size (bp)	Ref.
Used primers for detection	influenza A virus		M30F2/08	ATGAGYCTTYTAACCGAGGTCGAAACG	244	[2]
			M264R3/08	TGGACAAANCGTCTACGCTGCAG		
Used primers for typing	(A/H1N1) Pdm09		HKU-SWF	TGAGCTCAGTGTCATCATTTGA	174	[2]
			HKU-SWR	TGCTGAGCTTTGGGTATGAA		
	A/H3N2		H3A1F6	AAGCAGGGGATAATTCTATTAACC	1127	
			H3A1R1	GTCTATCATTCCCTCCCAACCATT		
Used primers for sequencing	A/H1N1 pdm09	*HA*	H1-F1	AGCAAAAGCAGGGGAAAATAAAAGC	1264	
			H1-R1	CCTACTGCTGTGAACTGTGTATTC		
			H1-F2	GGGAGAATGAACTATTACTGG	979	[2]
			H1-R2	AGTAGAAACAAGGGTGTTTTT		
	A/H1N1 pdm09	*NA*	N1-F1	AGCAAAAGCAGGAGTTTAAAATG	1099	
			N1-R1	CCTATCCAAACACCATTGCCGTAT		
			N1-F2	GGAATGCAGAACCTTCTTCTTGAC	1073	
			N1-R2	ATATGGTCTCGTATTAGTAGAAACAAGGAGTTTTTT		

**Table 2:** Distributions of clinical samples according to season, gender, and age.

		No. of Samples *n* (%)	Positive for Influenza A Virus *n* (%)	Positive for	
				A/H1N1pdm09 *n* (%)	A/H3N2 *n* (%)
Total		363	110 (30.3)	**68 (61.8)**	**42 (38.2)**
**Year**	2024	166 (45.7)	47 (28.3)	38 (80.8)	9 (19.2)
	2025	197 (54.3)	63 (31.9)	30 (47.6)	33 (52.4)
**Gender**	Male	176 (48.4)	45 (25.5)	29 (64.4)	16 (35.5)
	Female	187 (51.5)	65 (34.7) ^a^	39 (60) ^a^	26 (39.4)
**Age in years**	0–4	121 (33.3)	27 (22.3)	13 (48.1)	14 (51.8)
	5–14	88 (24.2)	29 (32.9)	20 (68.9)	9 (31.0)
	15–64	101 (27.8)	33 (32.6) ^b^	16 (48.4)	17 (51.5) ^b^
	≥65	53 (14.6)	21 (39.6)	19 (90.4)	2 (9.5)

Note: The presented data are shown in number (%). ^a^ Significantly higher (*p* < 0.05) than males. ^b^ Significantly higher (*p* < 0.05) than age groups 0–4, 5–14, and ≥65 years.

**Table 3:** Amino acid residues at key antigenic sites (130-loop, 190-helix, and 220-loop) in HA protein of influenza A(H1N1) pdm09 virus strains, highlighting sequence homology.

	130-Loop				190-Helix								220-Loop							
Mutation sites	134	135	136	138	188	189	190	191	192	193	194	195	221	222	223	224	225	226	227	228
A/California/7/2009	**F**	**P**	**K**	**S**	K	E	V	L	V	L	W	G	S	R	Y	S	K	K	F	K
A/Wisconsin/67/2022	**.**	**.**	**.**	**.**	.	.	.	.	.	.	.	.	.	.	.	.	.	.	.	.
A/Victoria/4897/2022	**.**	**.**	**.**	**.**	.	.	.	.	.	.	.	.	.	.	.	.	.	.	.	.
A/Riyadh/35/2024	**.**	**.**	**.**	**.**	.	.	.	.	.	.	.	.	.	.	.	.	.	.	.	.
A/Riyadh/40/2024	**.**	**.**	**.**	**.**	.	.	.	.	.	.	.	.	.	.	.	.	.	.	.	.
A/Riyadh/41/2024	**.**	**.**	**.**	**.**	.	.	.	.	.	.	.	.	.	.	.	.	.	.	.	.
A/Riyadh/145/2024	**.**	**.**	**.**	**.**	.	.	.	.	.	.	.	.	.	.	.	.	.	.	.	.
A/Riyadh/154/2024	**.**	**.**	**.**	**.**	.	.	.	.	.	.	.	.	.	.	.	.	.	.	.	.
A/Riyadh/169/2025	**.**	**.**	**.**	**.**	.	.	.	.	.	.	.	.	.	.	.	.	.	.	.	.
A/Riyadh/250/2025	**.**	**.**	**.**	**.**	.	.	.	.	.	.	.	.	.	.	.	.	.	.	.	.

Note. Dots indicate identical residues. The 130-loop (residues 134–138) is represented by the blue color; the 190-helix (residues 188–195) is represented by the green color; the 220-loop (residues 221–228) is represented by the red color.

## Data Availability

The datasets generated and analyzed during the current study are available from the corresponding author upon reasonable request. The sequence data reported in this study have been deposited in GenBank under accession numbers PV652976 to PV652982 for the *HA* gene and PV653584 to PV653590 for the *NA* gene.

## Supplementary Materials

The following supporting information can be downloaded at: https://www.mdpi.com/article/10.3390/vaccines13111111/s1, Table S1: List of A/H1N1 strains included in sequence and phylogenetic analysis; Figure S1: Alignment and comparison between A/Riyadh A/H1N1pdm09 strains and the reference strain (A/California/7/2009) of *HA* gene; Figure S2: Alignment and comparison between A/Riyadh A/H1N1pdm09 strains and the reference strain (A/California/7/2009) of *NA* gene.
