# Supplementary material for: Genetic Divergence of H1N1pdm09 in Saudi Arabia: Unveiling a Novel N-Glycosylation Site and Its Role in Vaccine Mismatch
Source: Vaccines (Basel). 2025 Oct 30;13(11):1111. doi: 10.3390/vaccines13111111 (PMC12656348; doi:10.3390/vaccines13111111)
Supplement: Supplementary file 1 [file vaccines-13-01111-s001.zip › vaccines-3898044-supplementary.pdf]

Table S1: **List of A/H1N1 strains included in sequence and phylogenetic analysis**

**No.**	**Strain**	**Origin**	**GenBank accession No.**			
			**H1**	**N1**	**Clade**	**Note**
**1.**	**A/Michigan/45/2015**	**USA**	**MK622940.**	**MK622934**	**6B.1**	**Vaccine strain**
**2.**	**A/Brisbane/02/2018**	**Australia**	**EPI1504919**	**EPI1504919**	**6B.1A.1**	**Vaccine strain**
**3.**	**A/India/3405/2017**	**India**	**EPI1161973**	**EPI1161972**	**6**	
**4.**	**A/Taiwan/80693/2018**	**Taiwan**	**EPI1281129**	**EPI1281128**	**6**	
**5.**	**A/Navarra/1985/2017**	**Spain**	**EPI1032468**	**EPI1281128**	**6B.1A.1**	
**6.**	**A/Aichi/190/2018**	**Japan**	**EPI1267660**	**EPI1267659**	**6B.1A.2**	
**7.**	**A/Darwin/102/2019**	**Australia**	**EPI1435917**	**EPI1435916**	**6B.1A.2**	
**8.**	**A/AbuDhabi/186/2017**	**UAE**	**EPI1180204**	**EPI1180203**	**6B.1A.3**	
**9.**	**A/Idaho/7/2018**	**USA**	**EPI1206974**	**EPI1206973**	**6B.1A.3**	
**10.**	**A/California/7/2009**	**USA**	**NC_026433**	**NC_026434**	**Protype 4**	**Vaccine strain**
**11.**	**A/Sydney/1167/2022**	**Australia**	**EPI2152082**	**EPI2152081**	**6B.1A.5a.2**	
**12.**	**A/Kyiv/359/2017**	**Ukraine**	**EPI1164245**	**EPI1164243**	**6B.1A.5b**	
**13.**	**A/CostaRica/4734/2020**	**Costa Rica**	**EPI1805661**	**EPI1805660**	**6B.1A.5b**	
**14.**	**A/Ireland/84630/2018**	**Ireland**	**EPI1354717**	**EPI1354718**	**6B.1A.6**	
**15.**	**A/Iceland/68/2019**	**Iceland**	**EPI1506821**	**EPI1506822**	**6B.1A.6**	
**16.**	**A/Paris/2186/2018**	**France**	**EPI1313521**	**EPI1313520**	**6B.1A.7**	
**17.**	**A/Norway/2967/2021**	**Norway**	**EPI1882571**	**EPI1882570**	**6B.1A.7**	
**18.**	**A/Israel/Q/504/2015**	**Israel**	**EPI697729**	**EPI697730**	**6B.2**	
**19.**	**A/Ningbo/75/2021**	**China**	**EPI1858137**	**EPI1858139**	**6B.2**	
**20.**	**A/Singapore/DMS1192/2011**	**Singapore**	**EPI634252**	**EPI634680**	**7**	
**21.**	**A/Ethiopia/198/2012**	**Ethiopia**	**EPI386042**	**EPI386041**	**8**	
**22.**	**A/SaudiArabia/129/2014**	**Saudi Arabi**	**MK246085**	**MK228959**	**6B.2**	
**23.**	**A/SaudiArabia/130/2014**	**Saudi Arabi**	**MK246086**	**MK228960**	**6B.2**	
**24.**	**A/SaudiArabia/131/2014**	**Saudi Arabi**	**MK246087**	**MK228961**	**6B.2**	

**25.**	**A/Saudi Arabia/67/2015**	**Saudi Arabi**	**MK246043**	**MK228917**	**6B.1**	
**26.**	**A/Saudi Arabia/69/2015**	**Saudi Arabi**	**MK246045**	**MK228919**	**6B.1**	
**27.**	**A/Saudi Arabia/03/2015**	**Saudi Arabi**	**MK246015**	**MK228889**	**6B.2**	
**28.**	**A/Saudi Arabia/04/2015**	**Saudi Arabi**	**MK246016**	**MK228890**	**6B.1**	
**29.**	**A/Jeddah/KFAFH3561/2015**	**Saudi Arabi**	**MF768811**	**MF768813**	**6B.1**	
**30.**	**A/Jeddah/KFAFH3274/2015**	**Saudi Arabi**	**MF768755**	**MF768757**	**6B.1**	
**31.**	**A/Jeddah/KFAFH1507/2015**	**Saudi Arabi**	**MF768779**	**MF768781**	**6B.1**	
**32.**	**A/Jeddah/KFAFH1335/2015**	**Saudi Arabi**	**MF768731**	**MF768733**	**6B.1**	
**33.**	**A/Jeddah/0267/2015**	**Saudi Arabi**	**MF768787**	**MF768789**	**6B.1**	
**34.**	**A/Jeddah/0114/2015**	**Saudi Arabi**	**MF768595**	**MF768597**	**6B.1**	
**35.**	**A/Wisconsin/67/2022**	**USA**	**EPI2224978**	**EPI2224977**	**5a.2a.1**	**Vaccine strain**
**36.**	**A/Victoria/4897/2022**	**Australia**	**EPI2319193**	**EPI2319192**	**5a.2a.1**	**Vaccine strain**
**37.**	**A/Iowa/06/2021**	**USA**	**EPI1941482**	**EPI1941481**	**6B.1A**	
**38.**	**A/Togo/44/2021**	**Togo**	**EPI1987609**	**EPI1987608**	**5a.1**	
**39.**	**A/Niger/8940/2021**	**Niger**	**EPI1951469**	**EPI1951468**	**5a.1**	
**~~40.~~**	**A/Cote_D_Ivoire/1973/2021**	**Cote d'Ivoir**	**EPI1885593**	**EPI1885592**	**5a.1**	
**41.**	**A/Murcia/10396/2021**	**Spain**	**EPI1987658**	**EPI1993865**	**5a.1**	
**42.**	**A/Argentina/3533/2022**	**Argentina**	**EPI2316317**	**EPI2316318**	**5a.1**	
**43.**	**A/Zambia/1139/2023**	**Zambia**	**EPI2901545**	**EPI2901543**	**5a.1**	
**44.**	**A/Connecticut/ATCC/01/2021**	**USA**	**EPI2754893**	**EPI2754892**	**5a.2a**	
**45.**	**A/Croatia/86715/2022**	**Croatia**	**EPI2134571**	**EPI2134572**	**5a.2a.1**	
**46.**	**A/Sao_Paulo/357189121/IAL/2023**	**Brazil**	**EPI2717449**	**EPI2717447**	**5a.2a.1**	
**47.**	**A/Indiana/02/2023**	**USA**	**EPI2619801**	**EPI2619795**	**5a.2a.1**	
**48.**	**A/Mountain_Ash/7986/2024**	**United Kingdom**	**EPI3596992**	**EPI3596994**	**5a.2a.1**	
**49.**	**A/Tasmania/386/2024**	**Australia**	**EPI3545286**	**EPI3545284**	**5a.2a.1**	

50.	A/Denmark/2329/2024	Denmark	EPI3626324	EPI3626323	5a.2a.1
51.	A/Georgia/31/2024	USA	EPI3246388	EPI3246387	5a.2a
52.	A/Auckland/80/2023	New Zealand	EPI2927397	EPI2927388	5a.2a
53.	A/British_Columbia/190/2022	Canada	EPI2127424	EPI2127423	5a.2a
54.	A/Estonia/KL262/2023	Estonia	EPI2639656	EPI2639657	5a.2a
55.	A/Minas_Gerais/10225/2024	Brazil	EPI3573271	EPI3573269	5a.2a.1
56.	A/Valparaso/31347/2023	Chile	EPI3179863	EPI3179862	5a.2a.1
57.	A/Rio_de_Janeiro/7705/2024	Brazil	EPI3401250	EPI3401248	5a.2a.1
58.	A/Galicia/35737931/2023	Spain	EPI3069030	EPI3069028	5a.2a.1
59.	A/Netherlands/01655/2023	Netherlands	EPI2982402	EPI2982401	5a.2a.1
60.	A/South_Africa/R04576/2022	South Africa	EPI2122017	EPI2122018	5a.2a.1
61.	A/Hong_Kong/2931/2024	China	EPI3743850	EPI3743849	5a.2a.1
62.	A/England/4780160/2024	United Kingdom	EPI3716712	EPI3716711	5a.2a.1
63.	A/Oman/CPHL_7247935/2024	Oman	EPI3736312	EPI3736311	5a.2a.1
64.	A/Nebraska/21/2024	USA	EPI3432381	EPI3432380	5a.2a.1
65.	A/Victoria/161/2024	Australia	EPI3236043	EPI3236042	5a.2a.1
66.	A/Latvia/01/066098/2024	Latvia	EPI3104025	EPI3104024	5a.2a.1
67.	A/Beijing/Chaoyang/SWL34/2023	China	EPI2669349	EPI2669348	5a.2a.1
68.	A/North_Dakota/22/2023	USA	EPI2499505	EPI2499500	5a.2a.1
69.	A/Bangladesh/4020/2021	Bangladesh	EPI1968732	EPI1968731	5a.2a
70.	A/Salamanca/44/2022	Spain	EPI2542034	EPI2550076	5a.2a.1
71.	A/Sydney/635/2023	Australia	EPI2719464	EPI2719460	5a.2a.1
72.	A/Saskatchewan/RV00407/2023	Canada	EPI2555710	EPI2555709	5a.2a.1
73.	A/Hubei/Xianan/SWL11444/2023	China	EPI2959682	EPI2959677	5a.2a.1
74.	A/Phra_Nakhon_Si_Ayutthaya/P2811/2023	Thailand	EPI2713317	EPI2713316	5a.2a
75.	A/AOMORI/27/2023	Japan	EPI2977131	EPI2977130	5a.2a.1

**76.**	**A/Peru/LAL/INS/183/2024**	**Peru**	EPI3763395	EPI3763394	**5a.2a.1**
**77.**	**A/Antananarivo/01843/2023**	**Madagascarr**	EPI2745337	EPI2762775	**5a.2a**
**78.**	**A/Surat_Thani/F263/2025**	**Thailand**	EPI3863330	EPI3863329	5a.2a.1
**79.**	**A/Tennessee/59/2022**	**USA**	EPI2349367	EPI2349366	5a.2a.1
**80.**	**A/Lisboa/124/2023**	**Portugal**	EPI2899921	EPI2899920	5a.2a.1
**81.**	**A/Panama/M272814/2024**	**Panama**	EPI3107920	EPI3107922	5a.2a.1
**82.**	**A/Eskilstuna/SE24/15080/2024**	**Sweden**	EPI3669587	EPI3669586	5a.2a.1
**83.**	**A/Badajoz/18662850/2024**	**Spain**	EPI3735570	EPI3735569	5a.2a.1

A

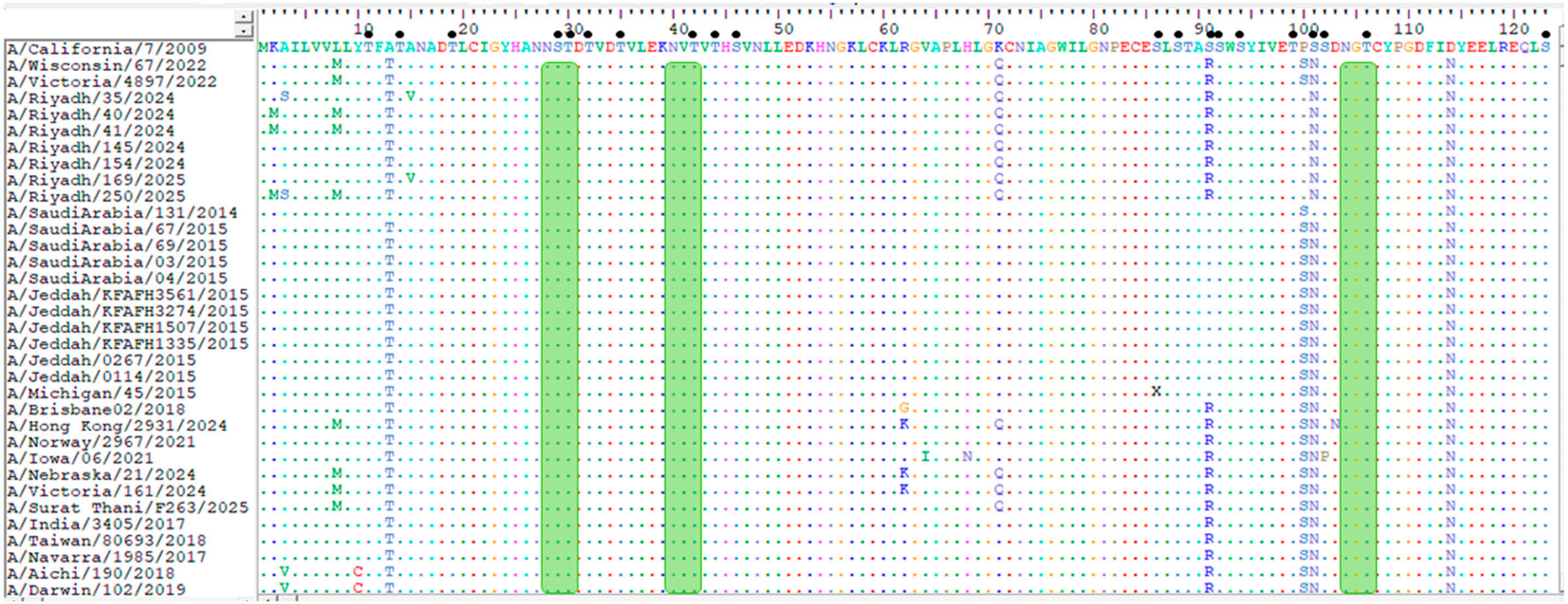
10
20
30
40
50
60
70
80
90
100
110
120
A/California/7/2009
A/Wisconsin/67/2022
A/Victoria/4897/2022
A/Riyadh/35/2024
A/Riyadh/40/2024
A/Riyadh/41/2024
A/Riyadh/145/2024
A/Riyadh/154/2024
A/Riyadh/169/2025
A/Riyadh/250/2025
A/SaudiArabia/131/2014
A/SaudiArabia/67/2015
A/SaudiArabia/69/2015
A/SaudiArabia/03/2015
A/SaudiArabia/04/2015
A/Jeddah/KFAFH3561/2015
A/Jeddah/KFAFH3274/2015
A/Jeddah/KFAFH1507/2015
A/Jeddah/KFAFH1335/2015
A/Jeddah/0267/2015
A/Jeddah/0114/2015
A/Michigan/45/2015
A/Brisbane02/2018
A/Hong Kong/2931/2024
A/Norway/2967/2021
A/Iowa/06/2021
A/Nebraska/21/2024
A/Victoria/161/2024
A/Surat Thani/F263/2025
A/India/3405/2017
A/Taiwan/80693/2018
A/Navarra/1985/2017
A/Aichi/190/2018
A/Darwin/102/2019

B

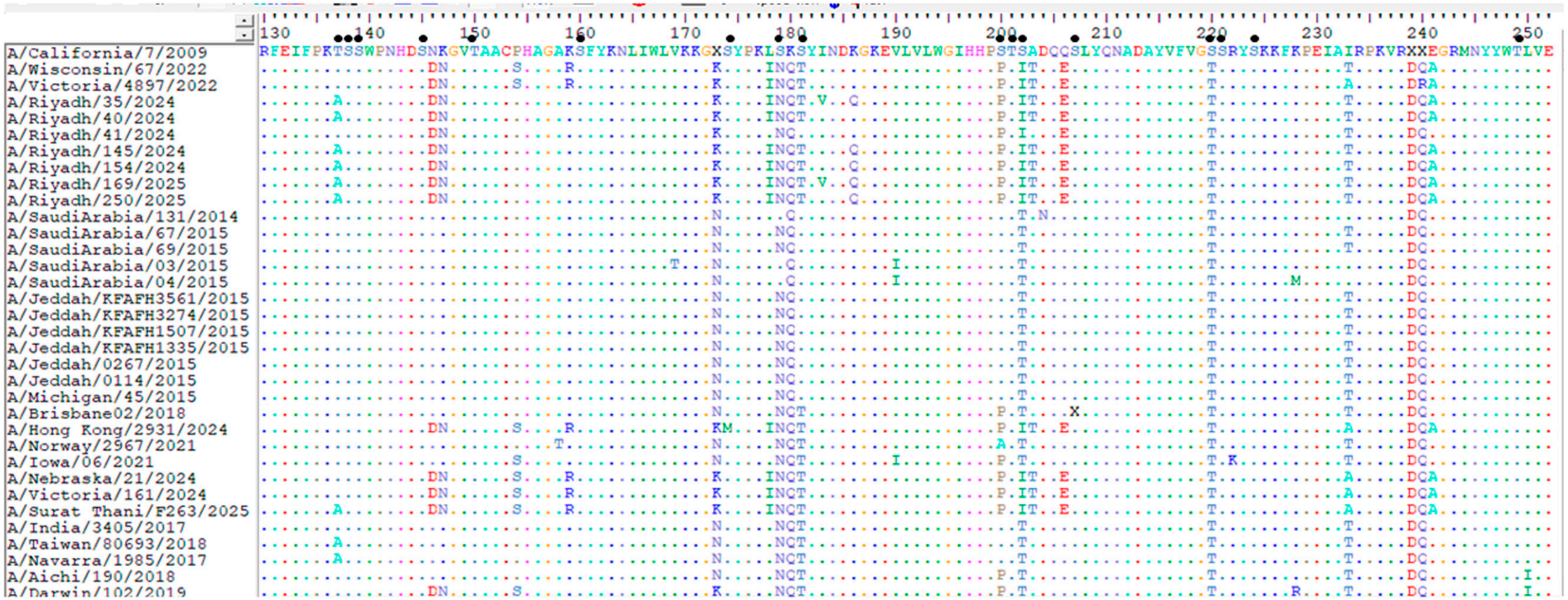
130
140
150
160
170
180
190
200
210
220
230
240
250
A/California/7/2009
A/Wisconsin/67/2022
A/Victoria/4897/2022
A/Riyadh/35/2024
A/Riyadh/40/2024
A/Riyadh/41/2024
A/Riyadh/145/2024
A/Riyadh/154/2024
A/Riyadh/169/2025
A/Riyadh/250/2025
A/SaudiArabia/131/2014
A/SaudiArabia/67/2015
A/SaudiArabia/69/2015
A/SaudiArabia/03/2015
A/SaudiArabia/04/2015
A/Jeddah/KFAFH3561/2015
A/Jeddah/KFAFH3274/2015
A/Jeddah/KFAFH1507/2015
A/Jeddah/KFAFH1335/2015
A/Jeddah/0267/2015
A/Jeddah/0114/2015
A/Michigan/45/2015
A/Brisbane02/2018
A/Hong Kong/2931/2024
A/Norway/2967/2021
A/Iowa/06/2021
A/Nebraska/21/2024
A/Victoria/161/2024
A/Surat Thani/F263/2025
A/India/3405/2017
A/Taiwan/80693/2018
A/Navarra/1985/2017
A/Aichi/190/2018
A/Darwin/102/2019

C

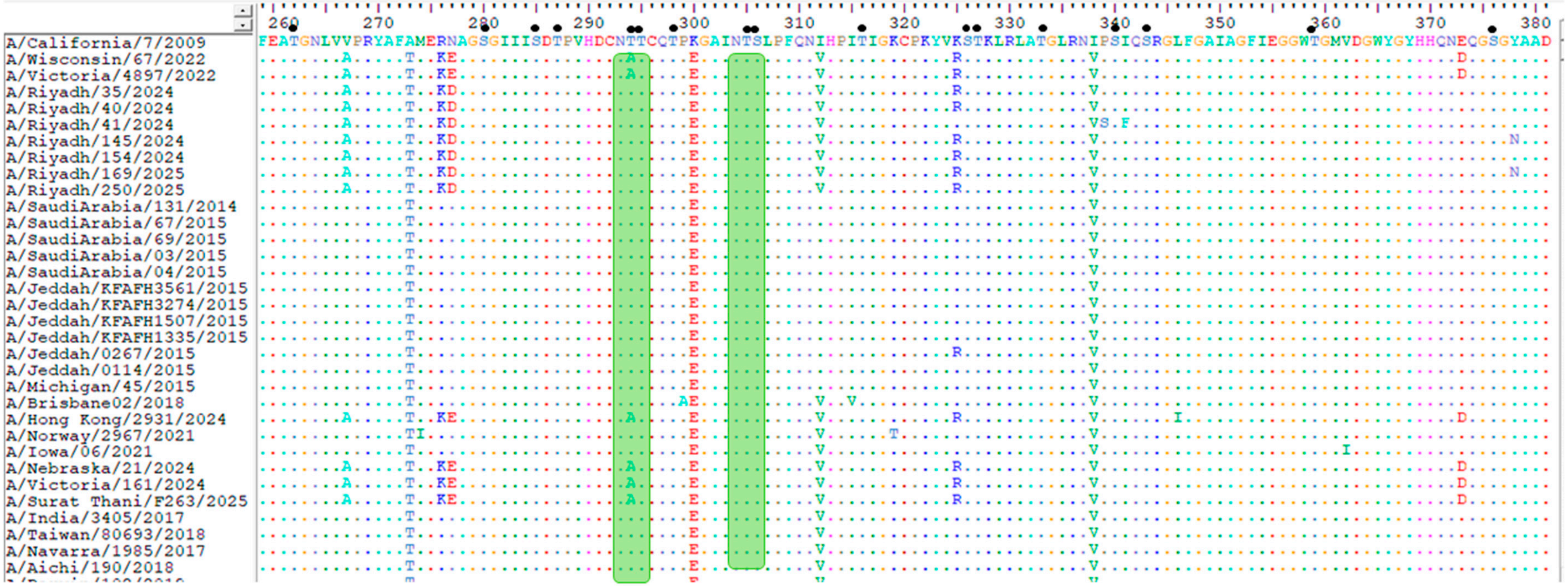
A/California/7/2009
A/Wisconsin/67/2022
A/Victoria/4897/2022
A/Riyadh/35/2024
A/Riyadh/40/2024
A/Riyadh/41/2024
A/Riyadh/145/2024
A/Riyadh/154/2024
A/Riyadh/169/2025
A/Riyadh/250/2025
A/SaudiArabia/131/2014
A/SaudiArabia/67/2015
A/SaudiArabia/69/2015
A/SaudiArabia/03/2015
A/SaudiArabia/04/2015
A/Jeddah/KFAFH3561/2015
A/Jeddah/KFAFH3274/2015
A/Jeddah/KFAFH1507/2015
A/Jeddah/KFAFH1335/2015
A/Jeddah/0267/2015
A/Jeddah/0114/2015
A/Michigan/45/2015
A/Brisbane02/2018
A/Hong Kong/2931/2024
A/Norway/2967/2021
A/Iowa/06/2021
A/Nebraska/21/2024
A/Victoria/161/2024
A/Surat Thani/F263/2025
A/India/3405/2017
A/Taiwan/80693/2018
A/Navarra/1985/2017
A/Aichi/190/2018

D

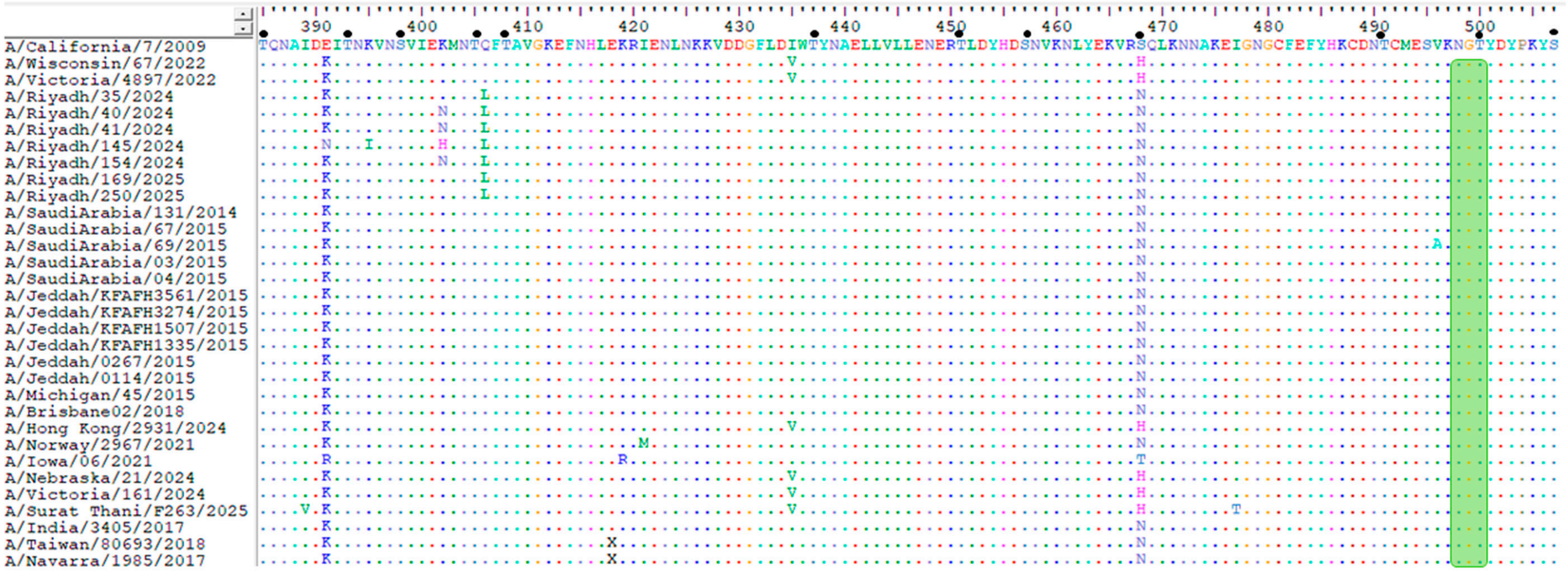

Figure S1. (A, B, C, and D), Alignment and comparison between A/Riyadh A/H1N1pdm09 strains and the reference strain (A/California/7/2009) of *HA* gene. Similar amino acids were presented by colored dots, while amino acid changes occurred and are shown in capitalized alphabetical order. Location of N-glycosylation site indicated in green rectangles and possible O-glycosylation site marked by black dots.

A

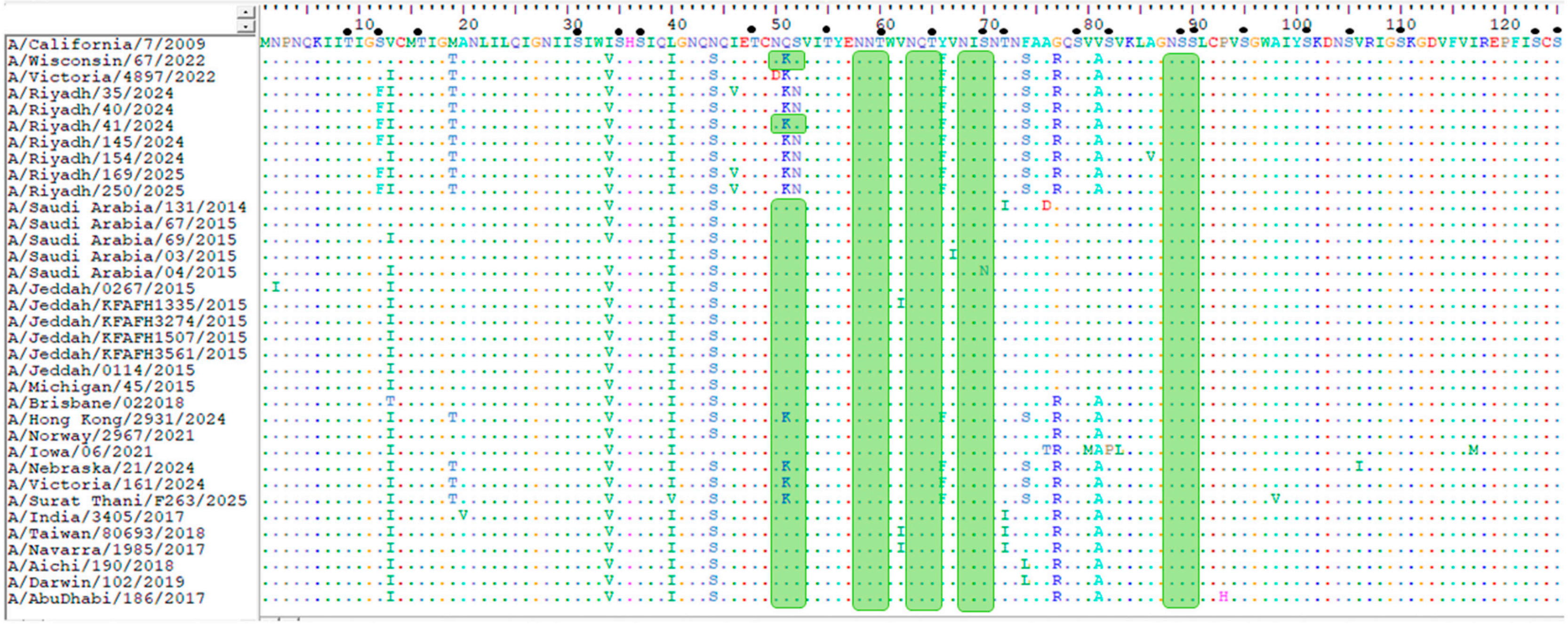

10 20 30 40 50 60 70 80 90 100 110 120
MNPNQKIITIGSVCMTIGMANLILQIGNIISIWISHSIQLGNQNQIETCNQSVITYENNTWVNQTYVNISNTNFAAGQSVVSVKLAGNSSLCPVSGWAIYSKDNSVRIGSKGDVFVIREPFISCS
A/California/7/2009
A/Wisconsin/67/2022
A/Victoria/4897/2022
A/Riyadh/35/2024
A/Riyadh/40/2024
A/Riyadh/41/2024
A/Riyadh/145/2024
A/Riyadh/154/2024
A/Riyadh/169/2025
A/Riyadh/250/2025
A/Saudi Arabia/131/2014
A/Saudi Arabia/67/2015
A/Saudi Arabia/69/2015
A/Saudi Arabia/03/2015
A/Saudi Arabia/04/2015
A/Jeddah/0267/2015
A/Jeddah/KFAFH1335/2015
A/Jeddah/KFAFH3274/2015
A/Jeddah/KFAFH1507/2015
A/Jeddah/KFAFH3561/2015
A/Jeddah/0114/2015
A/Michigan/45/2015
A/Brisbane/022018
A/Hong Kong/2931/2024
A/Norway/2967/2021
A/Iowa/06/2021
A/Nebraska/21/2024
A/Victoria/161/2024
A/Surat Thani/F263/2025
A/India/3405/2017
A/Taiwan/80693/2018
A/Navarra/1985/2017
A/Aichi/190/2018
A/Darwin/102/2019
A/AbuDhabi/186/2017

B

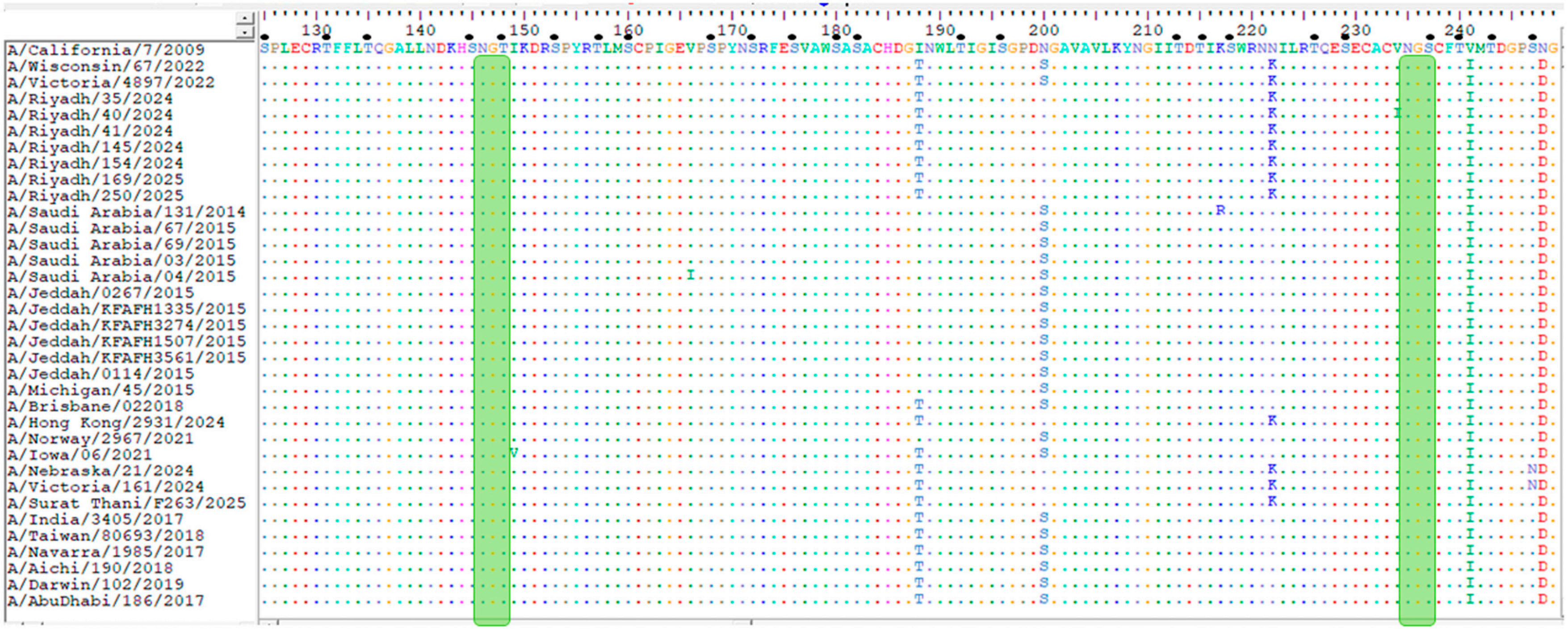
A/California/7/2009
A/Wisconsin/67/2022
A/Victoria/4897/2022
A/Riyadh/35/2024
A/Riyadh/40/2024
A/Riyadh/41/2024
A/Riyadh/145/2024
A/Riyadh/154/2024
A/Riyadh/169/2025
A/Riyadh/250/2025
A/Saudi Arabia/131/2014
A/Saudi Arabia/67/2015
A/Saudi Arabia/69/2015
A/Saudi Arabia/03/2015
A/Saudi Arabia/04/2015
A/Jeddah/0267/2015
A/Jeddah/KFAFH1335/2015
A/Jeddah/KFAFH3274/2015
A/Jeddah/KFAFH1507/2015
A/Jeddah/KFAFH3561/2015
A/Jeddah/0114/2015
A/Michigan/45/2015
A/Brisbane/022018
A/Hong Kong/2931/2024
A/Norway/2967/2021
A/Iowa/06/2021
A/Nebraska/21/2024
A/Victoria/161/2024
A/Surat Thani/F263/2025
A/India/3405/2017
A/Taiwan/80693/2018
A/Navarra/1985/2017
A/Aichi/190/2018
A/Darwin/102/2019
A/AbuDhabi/186/2017
130
140
150
160
170
180
190
200
210
220
230
240
SPLECRTFFLTQGALLNDKHSNGTIKDRSPYRTLMSCPIGEVPSPYNSRFESVAWSASACHDGINWLTIGISGPDNGAVAVLKYNGIITDTIKSWRNNILRTQESECACVNGSCFTVMTDGPSNG

C

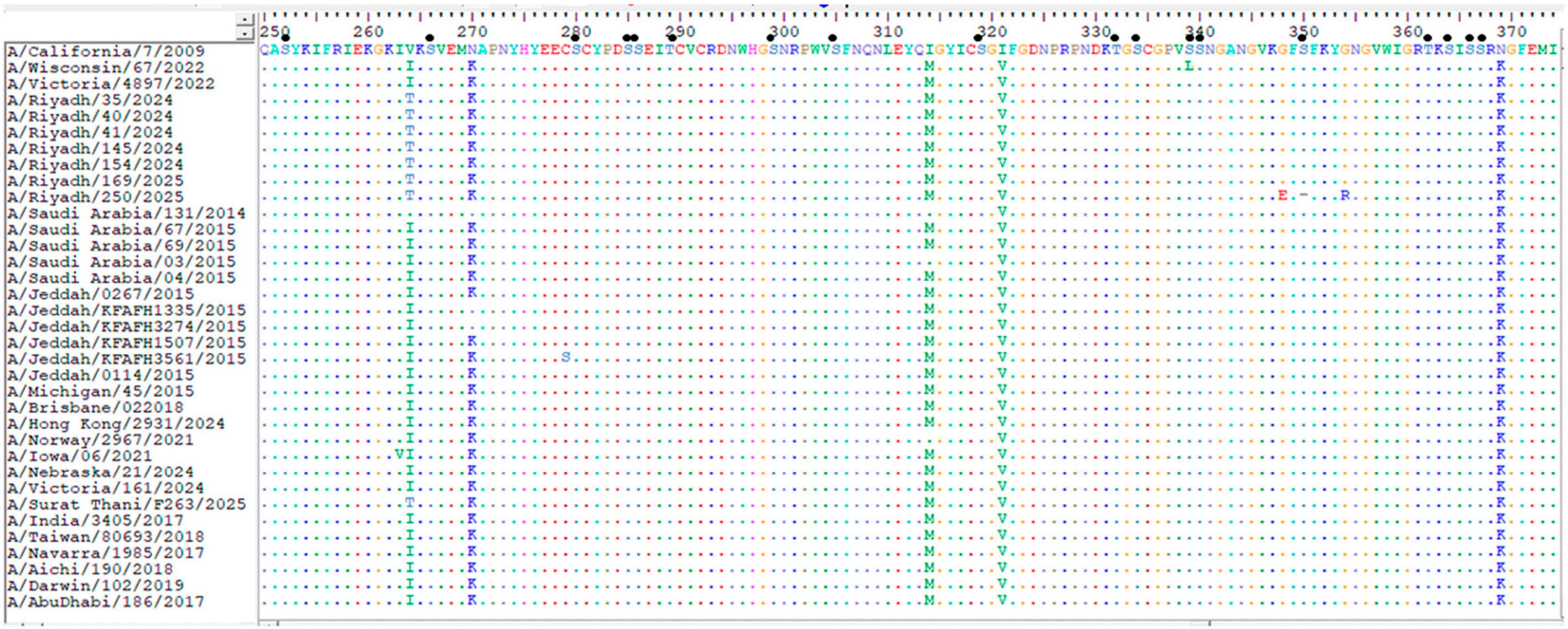
250
260
270
280
290
300
310
320
330
340
350
360
370
A/California/7/2009
A/Wisconsin/67/2022
A/Victoria/4897/2022
A/Riyadh/35/2024
A/Riyadh/40/2024
A/Riyadh/41/2024
A/Riyadh/145/2024
A/Riyadh/154/2024
A/Riyadh/169/2025
A/Riyadh/250/2025
A/Saudi Arabia/131/2014
A/Saudi Arabia/67/2015
A/Saudi Arabia/69/2015
A/Saudi Arabia/03/2015
A/Saudi Arabia/04/2015
A/Jeddah/0267/2015
A/Jeddah/KFAFH1335/2015
A/Jeddah/KFAFH3274/2015
A/Jeddah/KFAFH1507/2015
A/Jeddah/KFAFH3561/2015
A/Jeddah/0114/2015
A/Michigan/45/2015
A/Brisbane/022018
A/Hong Kong/2931/2024
A/Norway/2967/2021
A/Iowa/06/2021
A/Nebraska/21/2024
A/Victoria/161/2024
A/Surat Thani/F263/2025
A/India/3405/2017
A/Taiwan/80693/2018
A/Navarra/1985/2017
A/Aichi/190/2018
A/Darwin/102/2019
A/AbuDhabi/186/2017

D

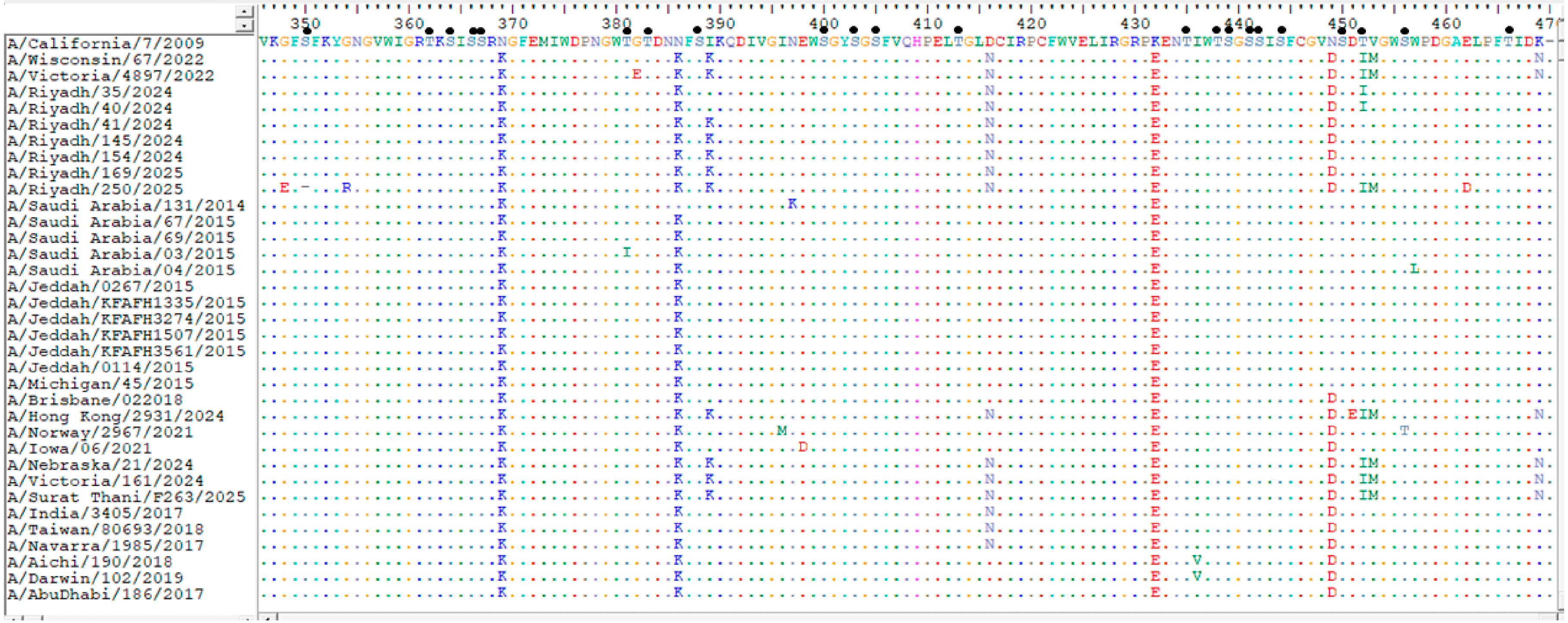

Figure S2. (A, B, C, and D), Alignment and comparison between A/Riyadh A/H1N1pdm09 strains and the reference strain (A/California/7/2009) of *NA* gene. Similar amino acids were presented by colored dots, while amino acid changes occurred and are shown in capitalized alphabetical order. Location of N-glycosylation site indicated in green rectangles and possible O-glycosylation site marked by black dots.
